# Phytochemicals and Bioactivities of *Zingiber cassumunar* Roxb

**DOI:** 10.3390/molecules26082377

**Published:** 2021-04-19

**Authors:** Ah-Reum Han, Hyunyoung Kim, Donglan Piao, Chan-Hun Jung, Eun Kyoung Seo

**Affiliations:** 1 Advanced Radiation Technology Institute, Korea Atomic Energy Research Institute (KAERI), Jeongeup-si, Jeollabuk-do 56212, Korea; arhan@kaeri.re.kr; 2 College of Pharmacy, Graduate School of Pharmaceutical Sciences, Ewha Womans University, Seoul 03760, Korea; fanet0106@naver.com (H.K.); parkdl@ehwain.net (D.P.); 3 Jeonju AgroBio-Materials Institute, Jeonju-si, Jeollabuk-do 54810, Korea; biohun@gmail.com

**Keywords:** *Zingiber cassumunar*, Zingiberaceae, phenylbutenoid, curcuminoid, essential oil, antioxidant, anti-inflammation, anticancer, neurotrophic activity, cosmeceutical property

## Abstract

*Zingiber cassumunar* Roxb. (Zingiberaceae), is an important medicinal plant known as “Plai (Phlai)” in Thailand, “Bangle” in Indonesia, and “Bulei” in China. Traditionally, this plant has been used to treat inflammation, pain, and respiratory problems. The rhizomes are the primary part of the plant that has been used for medicinal purposes due to their constituents with therapeutic properties, including phenylbutenoids, curcuminoids, and essential oils. Since the 1970s, many studies have been conducted on the phytochemicals and bioactivities of *Z. cassumunar* to establish fundamental scientific evidence that supports its use in traditional medicine. The accumulated biological studies on the extracts, solvent fractions, and constituents of *Z. cassumunar* have described their diverse medicinal properties, including antioxidant, anti-inflammatory, anticancer, neuroprotective/neurotrophic, cosmeceutical, and antifungal/antimicrobial bioactivities. In this review, we summarize information on the phytochemicals of *Z. cassumunar* and the bioactivities of its extracts and constituents.

## 1. Introduction

**Figure 1:**
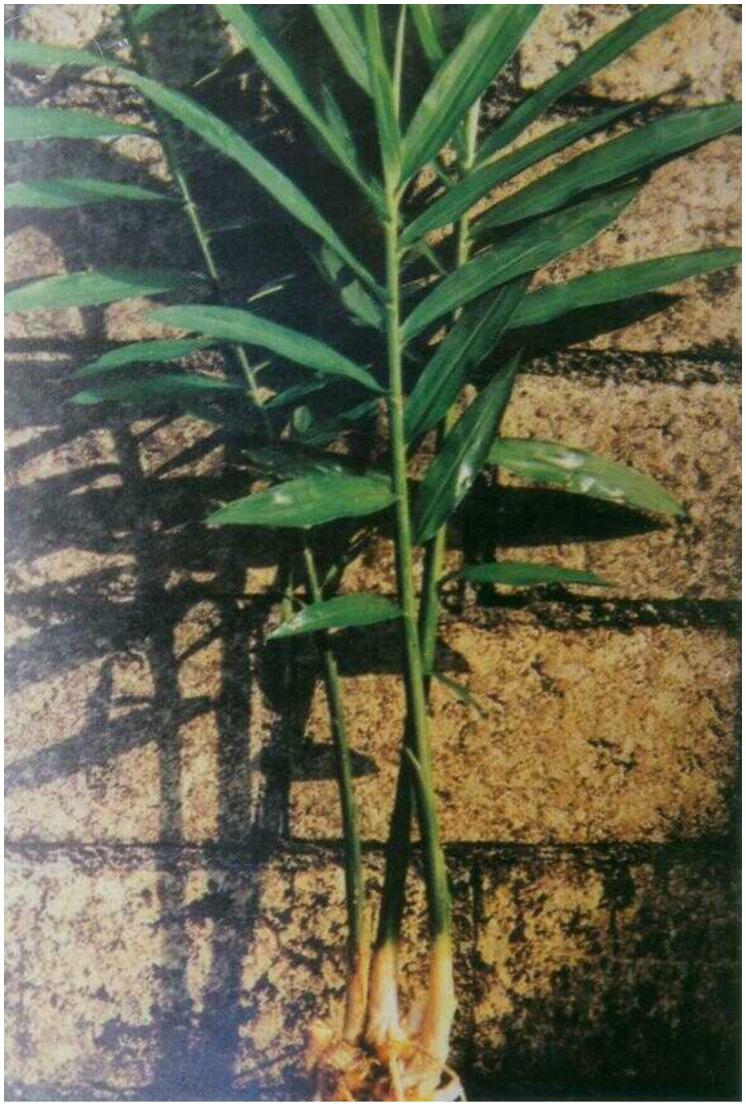
Photograph of a sample of *Z. cassumunar* collected from Surabaya, Indonesia, in 2001, which was identified by Professor Tri Windono (University of Surabaya, Indonesia).

*Zingiber cassumunar* Roxb. belongs to the family Zingiberaceae and is an herbaceous and perennial plant consisting of an underground part consisting of rhizomes (Figure 1). In Thailand, Indonesia, and other Asian countries, *Z. cassumunar* has traditionally been used as a medicinal plant in folk remedies for the treatment of various illness, such as inflammation, including arthritis, rheumatisms, sprains, respiratory problems such as asthma and cough, and pain caused by musculoskeletal, menstrual, or gastrointestinal disorders [1]. Several types of compounds in *Z. cassumunar* have been identified such as phenylbutenoids, curcuminoids, sesquiterpenoids, benzaldehydes, quinones, and essential oils containing monoterpenoids. Phenylbutenoids are characteristic compounds of this plant and have been isolated using various isolation technics [2,3,4,5,6,7,8,9,10,11,12,13,14], including silica gel or reverse phase column chromatography [2,3,4,5,6,7,8,9,10], recrystallization [3,4], preparative thin-layer chromatography [6], countercurrent chromatography [11,12], and preparative high-performance liquid chromatography [10,13,14]. Other constituents include curcuminoids, including a high content of curcumin as a yellow pigment in the rhizome [13,14,15,16,17], two quinones [3,4,18], phenolic compounds [13,17], and sesquiterpenoids such as (−)-β-sesquiphellandrene and zerumbone [12,19,20,21]. The essential oils included sabinene and terpinen-4-ol as major volatile terpenes [22,23,24,25]. The extracts and constituents of *Z. cassumunar* have diverse bioactivities, including antioxidant [21,26,27,28], anti-inflammatory [7,9,12,19,23,29,30,31,32,33,34,35,36,37,38,39], anticancer [8,13,15,40,41,42,43,44,45,46,47,48], neuroprotective/neurotrophic [14,19,20], cosmeceutical [17,21], and antifungal/antibacterial [22,23,24] activities. Although there has been a review of the clinical effects of various formulations using Plai (*Z. cassumunar*) on were pain relief, acne treatment, and antihistamine [1], there has been no previous report summarizing the accumulated studies in the literature on the phytochemicals and in vitro and in vivo biological properties of *Z. cassumunar*, including our previous studies that have contributed to discovering the chemical diversity and biological activities of this plant [8,9,15,21,25,26,27,28,29,30,31,32].

## 2. Phytochemicals from *Z. cassumunar*

### 2.1. Phenylbutenoids

Phenylbutenoids have been isolated mainly from *Z. cassumunar* [2,3,4,5,6,7,8,9,10,11,12,13,14] but have been found in other Zingiberaceae plants, including *Z. purpureum* [14,20], *Z. montanum* [22], which is known as “Bangle” or “cassumunar ginger,” *Z. neesanum* [33], and *Alpinia flabellate* [34]. Recently, the discovery of phenylbutenoids was reported for the plant of another family, *Dryopteris fragrans* (Dryopteridaceae) [35].

**Figure 2:**
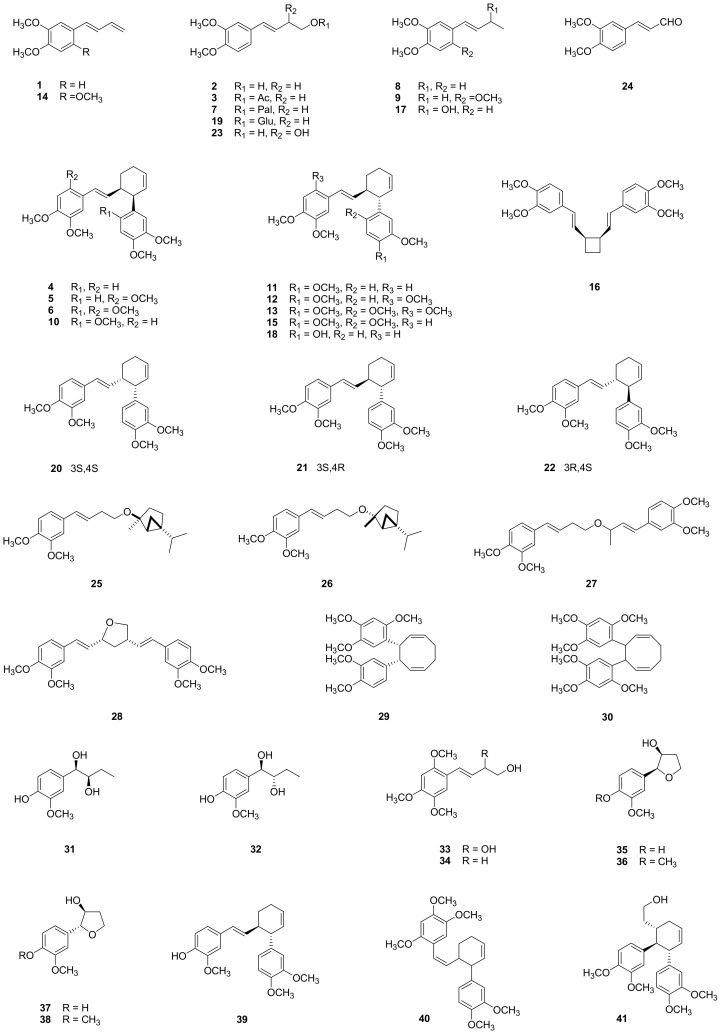
Structures of phenylbutenoids isolated from *Z. cassumunar*.

Barker et al. [2] discovered a phenylbutenoid, (*E*)-1-(3′,4′-dimethoxyphenyl)but-1,3-diene (**1**), in *Z. cassumunar*. Since then, a series of monomers and dimers were isolated from *Z. cassumunar*, and their structures were identified as (*E*)-4-(3′,4′-dimethoxyphenyl)but-3-en-l-ol (**2**); (*E*)-4-(3′,4′-dimethoxyphenyl)but-3-en-1-yl acetate (**3**); *cis*-3-(3′,4′-dimethoxyphenyl)-4-[(*E*)-3′′′,4′′′-dimethoxystyryl]cyclohex-1-ene (**4**); *cis*-3-(3′,4′-dimethoxyphenyl)-4-[(*E*)-2′′′,4′′′,5′′′-trimethoxystyryl]cyclohex-1-ene (**5**); and *cis*-3-(2′,4′,5′-trimethoxyphenyl)-4-[(*E*)-2′′′,4′′′,5′′′-trimethoxystyryl]cyclohex-l-ene (**6**) [3]. In a study that further characterized the constituents of *Z. cassumunar*, newly isolated phenylbutenoids, (*E*)-4-(3′,4′-dimethoxyphenyl)but-3-en-1-yl palmitate (**7**); (*E*)-4-(3′-4′-dime-thoxyphenyl)but-1-ene (**8**); (*E*)-4-(2′,4′,5′-trimethoxyphenyl)but-1-ene (**9**); *cis*-3-(2′,4′,5′-trimethoxyphenyl)-4-[(*E*)-3′′′,4′′′-dimethoxystyryl]cyclohex-l-ene (**10**); *trans*-3-(3,4-dimethoxyphenyl)-4-[(*E*)-3,4-dimethoxystyryl]cyclohex-1-ene (**11**); *trans*-3-(3′,4′-dimethoxyphenyl)-4-[(*E*)-2′′′,4′′′,5′′′-trimethoxystyryl]cyclohex-1-ene (**12**); and *trans*-3-(2′,4′,5′-trimethoxyphenyl)-4-[(*E*)-2′′′,4′′′,5′′′-trimethoxystyryl]cyclohex-l-ene (**13**) were reported [4]. Later, (*E*)-4-(2′,4′,5′-trimethoxyphenyl)but-1,3-diene (**14**) was isolated and identified by Tuntiwatchuntigul et al. [5]. The isolation of two more phenylbutenoid dimers, *trans*-3-(2′,4′,5′-trimethoxyphenyl)-4-[(*E*)-3′′′,4′′′-dimethoxystyryl]cyclohex-l-ene (**15**) and *cis*-1,2-bis[(*E*)-3,4-dimethoxystyryl]cyclobutane (**16**), was reported [6]. Phathong et al. [7] discovered (*E*)-4-(3′,4′-dimethoxyphenyl)but-3-en-2-ol (**17**) in *Z. cassumunar* [4]. In our previous phytochemical studies on *Z. cassumunar* [8,9], a new dimer type, *trans*-3-(4′-hydroxy-3′-methoxyphenyl)-4-[(*E*)-3′′′,4′′′-dimethoxystyryl]cyclohex-1-ene (**18**), and a new glycoside type, (*E*)-4-(3′,4′-dimethoxyphenyl)but-3-en-1-*O*-β-_D_-glucopyranoside (**19**), were isolated from the rhizomes of *Z. cassumunar* collected from Indonesia. Since the naturally occurring phenylbutenoid dimers were identified as racemic mixtures, but biological evaluations have been limited due to their low natural abundance, our research group carried out a total synthesis of optically active phenylbutenoid dimers and produced phenylbutenoid dimers with absolute configurations such as 3*S*-(3,4-dimethoxyphenyl)-4*S*-[(*E*)-3,4-dimethoxystyryl]cyclohex-1-ene (**20**); 3*S*-(3,4-dimethoxyphenyl)-4*R*-[(*E*)-3,4-dimethoxystyryl]cyclohex-1-ene (**21**); and 3*R*-(3,4-dimethoxyphenyl)-4*S*-[(*E*)-3,4-dimethoxystyryl]cyclohex-1-ene (**22**) [30]. The circular dichroism data for these compounds, for which their absolute configurations were established, have provided an excellent reference for determining the stereochemistry of phenylbutenoid dimers. There have also been reports on the preparative purification of phenylbutenoids from *Z. cassumunar* by countercurrent chromatography (CCC) [11,12]. Compounds **1** (175 mg) and **3** (150 mg) at more than 95% purity were successfully separated from the light petroleum extract of *Z. cassumunar* (600 g) using upright CCC in a single step, with the solvent system of light petroleum‒ethanol‒dimethyl ether‒water (5:4:2:1, *v*/*v*) [11]. This research group further performed elution-extrusion CCC using the *n*-hexane‒ethyl acetate‒methanol‒water (1:1:1:1, *v*/*v*) system for ethanol extraction from this plant, thereby producing milligram-amounts of four phenylbutenoids, (*E*)-4-(3′,4′-dimethoxyphenyl)but-1,2-diol (**23**); (*E*)-4-(3′,4′-dimethoxyphenyl)propenal (**24**); **2;** and **3,** which were more than 90% pure, and of a mixture of **4** and **11** [12]. A pair of diastereoisomers (**4** and **11**) was purified to the (+/−)-*trans* form and (+/−)-*cis* form at more than 98% pure via second-step separation by CCC. In the phytochemical study of *Z. cassumunar* by Nakamura et al. [13], six new phenylbutenoids were isolated and named phlains I‒VI (**25**‒**30**). Subsequently, Matsuda et al. [10] reported the isolation and identification of eight new compounds, cassumunols A‒H (**31**‒**38**). In the high-performance liquid chromatography/electrospray tandem mass spectroscopy (MS) analyses of phenylbutenoid dimers from *Z. cassumunar* by [36], two new structures were tentatively identified as *trans*-3-(3′,4′-dimethoxyphenyl)-4-[(*E*)-4′′′-hydroxy-3′′′-methoxystyryl]cyclohex-1-ene (**39**) and 3-(3′,4′-dimethoxyphenyl)-4-[(*Z*)-2′′′,4′′′,5′′′-trimethoxystyryl]cyclohex-1-ene (**40**). From Indonesian ginger, Bangle (*Z. purpureum*), a new phenylbutenoid dimer was isolated and identified as banglenol A (**41**) [14]. These compounds are detailed in Figure 2.

### 2.2. Other Compounds

**Figure 3:**
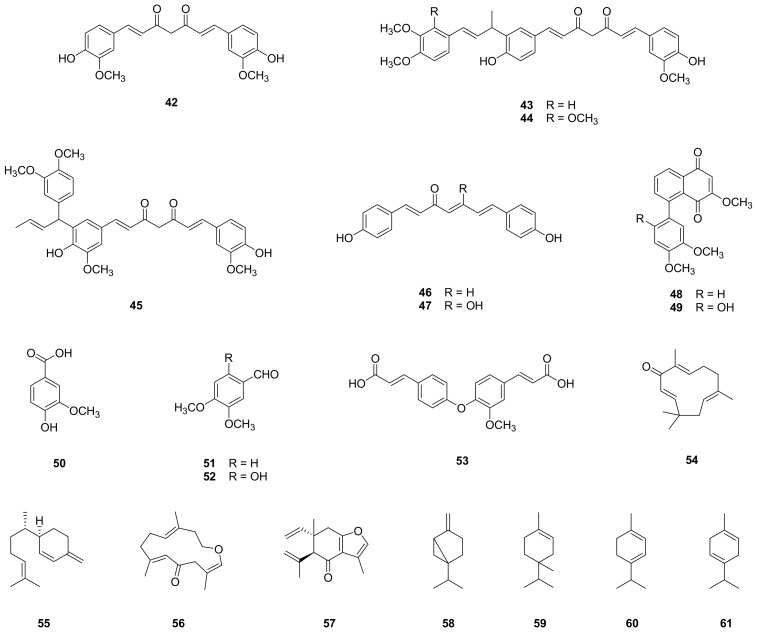
Structures of other compounds isolated from *Z. cassumunar*. Curcuminoids, **42**–**47**; quinones, **48** and **49**; phenolic compounds, **50**–**53**; sesquiterpenoids, **54**–**57**; and monoterpenoids, **58**–**61**.

Curcumin (**42**) is a major compound that was isolated from the rhizomes of *Z. cassumunar* with a yield of more than 0.2% by weight [13,15]. The related congeners, cassumunins A‒C (**43**‒**45**), were isolated from this plant by Masuda et al. [16]. Other curcumins, (1*E*,4*E*,6*E*)-1,7-Bis(4-hydroxyphenyl)-1,4,6-heptatrien-3-one (**46**) and bisdeoxycurcumin (**47**), were found in the rhizomes of *Z. cassumunar* [17]. In addition, two new curcuminoids, neocassumunarin A (**48**) and neocassumunarin B (**49**), were found in Indonesian ginger, Bangle (*Z. purpureum*) [14]. Two quinones were found in the rhizomes of *Z. cassumunar* and were identified as 2-methoxy-8(3,4-dimethoxyphenyl)-1,4-naphthoquinone (cassumunaquinone 1, **48**) and 2-methoxy-8(2,4,5-trimethoxyphenyl)-1,4-naphthoquinone (cassumunaquinone 2, **49**) [3,4,18]. There are other phenolic compounds in *Z. cassumunar*, such as vanillic acid (**50**); 3,4-dimethoxybenzaldehyde (**51**); 2,4,5-trimethoxybenzaldehyde (**52**); and 1-feruloyloxy cinnamic acid (**53**) [13,17]. As for the identification of sesquiterpenoids, zerumbone (**54**) [37,38] and β-sesquiphellandrene (**55**) [13] were isolated from the rhizomes of *Z. cassumunar*, and the essential oil extracted from the leaves of *Z. cassumunar* has been reported to contain 1(10),4-furanodien-6-one (**56**); curzerenone (**57**); and β-sesquiphellandrene [38]. Pharmaceutical or cosmeceutical products using essential oil from the rhizomes of *Z. cassumunar* have been developed; thus, the chemical composition of essential oil has been analyzed using usually gas chromatography-mass spectrometry (GC–MS). Monoterpenoids, sabinene (**58**), and terpinene-4-ol (**59**) were identified as major constituents [39] in rhizome essential oil, and α-terpinene (**60**), and γ-terpinene (**61**) were also discovered [22,40]. In addition to these components, the essential oil produced by steam contained approximately 25% phenylbutenoids, and oil extracted with light petroleum contained approximately 46% phenylbutenoids [41]. These compounds are presented in Figure 3.

## 3. Bioactivities of Extracts and Compounds from *Z. cassumunar*

Table 1 summarizes the various bioactivities of the extracts, solvent fractions, and compounds from *Z. cassumunar* that have been reported. Their pharmacological and nutraceutical properties as derived from the results of the bioassay, in vitro mechanism of action, or in vivo experiments are described in detail below.

### 3.1. Antioxidant Activities

It was discovered that curcuminoids isolated from *Z. cassumunar*, cassumunin A (**43**), and cassumunin B (**44**), prevent the H_2_O_2_-induced decrease in cell viability of thymocytes and protect living cells that are suffering from H_2_O_2_-induced oxidative stress [42]. The essential oil from *Z. cassumunar* contains three major components, sabinene, terpinen-4-ol, and (*E*)-1-(3,4-dimethyoxyphenyl)but-1,3-diene, which exhibited antioxidant activity upon scavenging the 2,2′-azinobis-(3-ethylbenzothiazoline-6-sulfonic acid (ABTS) cation radical and demonstrated H_2_O_2_ scavenging activity when emissions of dichlorodihydrofluorescein-fluorescence were reduced within a monocyte cell line (U937) [38]. In a study on the evaluation of solvent fractions of *Z. cassumunar* on in vitro antioxidant and α-glucosidase inhibitory assay [43], the chloroform fraction showed 2,2-diphenyl-1-picrylhydrazyl (DPPH) radical scavenging activity with an IC_50_ value of 78.19 μg/mL, while the hexane fraction demonstrated H_2_O_2_ scavenging activity with an IC_50_ value of 34.40 μg/mL and α-glucosidase inhibitory activities with an IC_50_ value of 61.02 μg/mL. The ethanol extract of the *Z. cassumunar* rhizome with 7% curcumin content was evaluated for its effect on superoxide dismutase (SOD) enzyme activity in rats fed a high-fat diet (HFD), which resulted in increased SOD activity upon oral administration of the extract (400 mg/kg, o.p.) in rats fed an HFD, compared to the control group and HFD group, which indicated that the *Z. cassumunar* rhizome has antioxidant activity and an ameliorate reactive oxygen species effect caused by HFD [44]. Therefore, in vitro and in vivo studies demonstrated that curcuminoids and essential oil from *Z. cassumunar* have strong antioxidant activities.

### 3.2. Anti-Inflammatory Activities

The anti-inflammatory activities of the extracts and constituents of *Z. cassumunar* were investigated in carrageenin-induced edema in rats and acetic acid-induced vascular permeability and writhing symptoms in mice [46]. Oral administration of methanol extract (3 g/kg) exhibited anti-inflammatory activity against edema and reduced the number of writhes induced by acetic acid. Oral administration of methanol extract (1 g/kg), ether-soluble fraction (1.3 g/kg), *n*-hexane-soluble fraction (0.2 g/kg), and (*E*)-1-(3′,4′-dimethoxyphenyl)but-1-ene (**8**) (0.016 g/kg) impacted vascular permeability induced by acetic acid in mice. In an assessment of the topical anti-inflammatory activities of the essential oil of *Z. cassumunar* rhizomes and its major components [40], a topical application of the essential oil inhibited the development of edema induced by carrageenan with an ID_50_ value of 22 mg/paw, and an ID_50_ value of (*E*)-1-(3′,4′-dimethoxyphenyl)but-1-ene (**8**) was estimated to be 3 mg/paw (diclofenac, ID_50_ 6 mg/paw). Terpine-4-ol (**59**) and α-terpinene (**60**) showed approximately 40% and 30% inhibition at the highest dose tested (6 mg/paw), respectively. Other tested compounds, sabinene (**58**) and γ-terpinene (**61**), were inactive. In addition, the hexane fraction of *Z. cassumunar* removing the essential oil was tested in the model of 12-*O*-tetradecanoylphorbol-13-acetate(TPA)-induced ear edema in rats and showed an effect with an ID_50_ value of 854 μg/ear [48]. The isolates, (*E*)-4-(3′,4′-dimethoxyphenyl)but-3-en-1-ol (**2**), (*E*)-4-(3′,4′-dimethoxyphenyl)but-3-enyl acetate (**3**); *cis*-3-(3′,4′-dimethoxyphenyl)-4-[(*E*)-3′′′,4′′′-dimethoxystyryl]cyclohex-1-ene (**4**); *cis*-3-(3′,4′-dimethoxyphenyl)-4-[(*E*)-2′′′,4′′′,5′′′-trimethoxystyryl]cyclohex-1-ene (**5**); and *cis*-3-(2′,4′,5′-trimethoxyphenyl)-4-[(*E*)-2′′′,4′′′,5′′′- trimethoxystyryl]cyclohex-1-ene (**6**) from this fraction exerted topical anti-inflammatory activities with ID_50_ values of 47, 62, 21, 20, and 2 μg/ear, respectively (diclofenac, ID_50_ 61 μg/ear). Further investigation of the anti-inflammatory activity of (*E*)-1-(3′,4′-dimethoxyphenyl)but-1,3-diene (**1**) using the in vivo and in vitro model suggested the effect of this compound on cyclooxygenase (COX) and lipoxygenase pathways [55]. This compound inhibited the rat ear edema induced by ethyl phenylpropiolate (EPP), arachidonic acid (AA), and TPA with IC_50_ values of 21, 60, and 660 nmol/ear. The rat paw edema induced by carrageenan was also inhibited by **1** with IC_50_ at 3 h of 22 μmol/paw. Compound **1** inhibited the platelet aggregation induced by collagen, adenosine diphosphate (ADP), AA, and the platelet-activating factor (PAF) with IC_50_ values of 0.35, 4.85, 0.94, and 1.14 mM. The anti-inflammatory activity of (*E*)-1-(3′,4′-dimethoxyphenyl)but-3-en-2-ol (**17**) has been investigated in several in vivo models [7]. Compound **17** reduced carrageenin-induced paw edema in rats by 83.9% at a dose of 300 mg/kg. In a test of the effect of **17** on carrageenin-induced rat pleurisy, **17** showed inhibitory activities against the formation of pleural exudate (52.3%) in the accumulation of leukocytes in the pleural exudate (56.5%), and in prostaglandin E_2_-like substances that were present in the inflamed exudate (48.8%). Compound **17** exhibited slight inhibitory effects on adjuvant-induced arthritis in primary (15.8%) and secondary lesions (injected [14.5%] and no injected paws [20.0%]). The analgesic properties of **17** were tested using an acetic acid-induced writhing response in mice and tail-flick test in rats, which exerted inhibitory activities on the writhing response and tail-flick response to radiant heat, with inhibitions of 52.0% and 11.7% at a dosage of 300 mg/kg (i.g.). Compound **17** showed an antipyretic effect by reducing the rectal temperature at a low dose of 75 mg/kg (i.g.) in yeast-induced hyperthermia in rats. In our previous study on the anti-inflammatory effects of phenylbutenoids from *Z. cassumunar* [9], four compounds, *trans*-3-(3,4-dimethoxyphenyl)-4-[(E)-3,4-dimethoxystyryl]cyclohex-1-ene (**11**); *trans*-3-(4′-hydroxy-3′-methoxyphenyl)-4-[(*E*)-3′′′,4′′′-dimethoxystyryl]cyclohex-1-ene (**18**); (*E*)-4-(2′,4′,5′-trimethoxyphenyl)but-1,3-diene (**14**); and (*E*)-1-(3′,4′-dimethoxyphenyl)but-1,3-diene (**1**) demonstrated inhibitory activities with IC_50_ values of 2.71, 3.64, 14.97, and 20.68 μM, respectively (celecoxib; IC_50_ 0.52 nM), in the COX-2 inhibitory assay by measuring prostaglandin E_2_ (PGE_2_) production in lipopolysaccharide (LPS)-stimulated mouse macrophage RAW264.7 cells. Another study examined the inhibitory effects of the constituents of *Z. cassumunar* on LPS-induced nitric oxide (NO) production in mouse peritoneal macrophages [13]. Compounds, plain I (**25**); plain III (**27**); plain VI (**30**); (*E*)-1-(3′,4′-dimethoxyphenyl)but-1,3-diene (**1**); (*E*)-4-(2′,4′,5′-trimethoxyphenyl)but-1,3-diene (**14**); (*E*)-4-(2′,4′,5′-trimethoxyphenyl)but-1-ene (**9**); cassumunaquinone 2 (**49**); curcumin (**42**); and β-sesquiphellandrene (**55**) exhibited inhibitory effects with IC_50_ values of 24, 24, 50, 69, 83, 31, 47, 11, and 52 μM, respectively (caffeic acid phenyl ester; IC_50_ 16 μM). In a study comparing the anti-inflammatory effects of a phenylbutenoid-rich fraction of *Z. cassumunar* with four individual phenylbutenoids and other crude extracts according to different extraction methods [45], the phenylbutenoid-rich fraction exhibited the strongest inhibitory activity on LPS-induced NO production in murine macrophage RAW264.7 cells. The phenylbutenoid-rich fraction prepared by one-step silica–gel column chromatography on the hexane extract exhibited an NO inhibitory effect with an IC_50_ value of 4.6 μg/mL, whereas hexane extracted under reflux and the essential oil obtained by a hydrodistillation inhibited NO production with IC_50_ values of 11.9 and 21.5μg/mL, respectively. The compounds (*E*)-4-(3,4-dimethoxyphenyl)but-3-en-l-ol (**2**); (*E*)-4-(3,4-dimethoxyphenyl)but-3-en-l-yl acetate (**3**); (*E*)-1-(3,4-dimethoxyphenyl)but-1,3-diene (**1**); and (*E*)-3-(3,4-dimethoxyphenyl)-4-[(*E*)-3,4-dimethoxystyryl]cyclohex-1-ene (**4** or **11**) displayed IC_50_ values of 211.1, 86.8, 56.3, and 39.7 μM, respectively (caffeic acid phenyl ester; IC_50_ 5.6 μM). In an assessment of the anti-inflammatory effects of the constituents of *Z. cassumunar* in human dental pulp cells [49], *cis*-3-(3′,4′-dimethoxyphenyl)-4-[(*E*)-3”‘,4”‘-dimethoxystyryl]cyclohex-1-ene (**4**); *cis*-3-(2′,4′,5′-trimethoxyphenyl)-4-[(E)-2”‘,4”‘,5”‘-trimethoxystyryl]cyclohex-1-ene (**6**); and (*E*)-1-(3′,4′-dimethoxyphenyl)but-1,3-diene (**1**) reduced the LPS-induced PGE2 level and COX-2 expression in human dental pulp cells.

*Z. cassumunar* was traditionally used to release pain in osteoarthritis and rheumatoid arthritis. Two constituents of this plant, c*is*-3-(2′,4′,5′-trimethoxyphenyl)-4-[(*E*)-2′’’,4′’’,5′’’-trimethoxystyryl]cyclohex-l-ene (**5**) and (*E*)-4-(3′,4′-dimethoxyphenyl)but-3-en-l-ol (**2**), each at a concentration of 100 μM, were found to possess chondroprotective effects against cytokine-induced cartilage degradation in the explant culture [50]. These two compounds inhibited the release of sulfated glycosaminoglycans and hyaluronic acid induced by Interleukin-1β (IL-1β) and suppressed IL-1β-induced loss of collagen and uronic acid contents from cartilage explants. The activities of matrix metalloproteinase (MMP)-2 and MMP-13 induced by IL-1β were reduced by 5 and 7. In a later study by Chaiwongsa et al. [51], (*E*)-4-(3′,4′-dimethoxyphenyl)but-3-en-l-ol (**2**) was found to downregulate the expression of MMPs (MMP-1, -2, -3, and -13) induced by IL-1β in a human synovial fibroblast SW982 cell line. Increases in the expressions of IL-1β and the IL-1β-converting enzyme were also inhibited by compound **2**.

The rhizomes of *Z. cassumunar* were used as an anti-asthmatic drug in Thai traditional medicine. There have been reports on the pharmacokinetic studies of (*E*)-4-(3′,4′-dimethoxyphenyl)but-3-en-1-ol (**2**), which has anti-inflammatory action [56,57]. The pharmacokinetics of **2** were studied in rats and monkeys using the in situ intestinal loop technique for asthmatic treatment [23]. The absorption of this compound reached its maximum at approximately 1 h after oral administration in rats and also exhibited an elimination half-life of approximately 2–2.3 h in both animal models. In addition, the pharmacokinetic profiles of **2**, including absorption, tissue distribution, and route of elimination, in male Wistar rats were also examined [24]. The compound showed good absorption, which reached its maximum at approximately 0.15 h after drug administration, and an excellent ratio of tissue to plasma that ranged from 1 to 1,000 in organs at 1‒4 h after drug administration. Less than 1% of **2** was detected in feces and urine. The ethanol extract of *Z*. *cassumunar* (5–100 μg/mL) significantly inhibited total mucin production, including MUC2 and MUC5AC mRNA and proteins induced by phorbol12-myristate 13-acetate (PMA) in human airway epithelial NCI-H292 cells [47]. The extracts also inhibited phosphorylation of extracellular signal-regulated kinase but not JNK and p38 in PMA-stimulated NCI-H292 cells. In the follow-up study [52], the ethanol extract and (*E*)-4-(3′,4′-dimethoxyphenyl)but-3-en-1-ol (**2**) from *Z. cassumunar* inhibited pro-MMP-9 cleavage by MMP-9 in asthma induced by house dust mite allergens. The extracts (100 mg/mL) and compound D (50 and 100 mg/mL) attenuated the PMA-induced MMP-9 gene and expression in NCI-H292 cells. Recently, the analysis of molecular interactions between two constituents of Z. *cassumunar* and a protein target, the 5-lipoxygenase (5-LO) enzyme, involved with asthma symptoms, were studied using molecular docking and molecular dynamics simulations [53]. (*E*)-1-(3′,4′-Dimethoxyphenyl)buta-1,3-diene (**1**) and (*E*)-4-(3′,4′-dimethoxyphenyl)but-3-en-1-ol (**2**) bound at the same catalytic site of its natural substrate (AA) on the 5-LO enzyme. The binding energy calculations of the 5-LO complex with **1**, **2**, and zileuton (anti-asthma agent) were −29.15, −26.83, and −29.40 kcal/mol, respectively, which supports the competition between the **1** and **2** substrate inhibitors, which was the same as zileuton [53]. Overall, these accumulated results indicated that the extracts and components of the *Z. cassumunar* possess potential value in the prevention and treatment of various diseases related to inflammation.

### 3.3. Anticancer Activities

Our research group has long carried out a series of studies on the anticancer activities of various components of *Z cassumunar*—from extracts to isolates [8,15,40,41,42,43,44,45,46,47]. The chloroform-soluble fraction of *Z. cassumunar* was found to have cytotoxicity against two human cancer cell lines (A549, lung; SNU-638, stomach) with IC_50_ values of 18.5 and 11.3 µg/mL, respectively [25]. Bioassay-guided fractionation of this fraction led to the isolation of several phenylbutenoids, and the cytotoxicities of these compounds have been evaluated against several human cancer cell lines (A549, lung; Col2, colon; SNU-638, stomach; HT-1080, fibrosarcoma) [8,15]. Compounds *trans*-3-(3,4-dimethoxyphenyl)-4-[(E)-3,4-dimethoxystyryl]cyclohex-1-ene (**11**) and *trans*-3-(4′-hydroxy-3′-methoxyphenyl)-4-[(*E*)-3′′′,4′′′-dimethoxystyryl]cyclohex-1-ene (**18**) exhibited moderate cytotoxicity against all tested human cancer cell lines, whereas (*E*)-1-(3′,4′-dimethoxyphenyl)but-1,3-diene (**1**) showed significant cytotoxicity against HT-1080 cells (IC_50_ value of 7.9 µM) in a selective manner. In a further mechanism study on the antiproliferative effect of **2** in A549 cells [26], *trans*-3-(3,4-dimethoxyphenyl)-4-[(E)-3,4-dimethoxystyryl]cyclohex-1-ene (**11**) induced G0/G1 phase cell cycle arrest by downregulating the expression of cyclin-dependent kinases (CDKs) and cyclins and by suppressing CDK activity via the induction of p21 expression. In addition, our research group has screened the P-glycoprotein (P-gp) inhibitory activities of four solvent fractions (hexanes, chloroform, *n*-butanol, and aqueous) of Indonesian medicinal plants in P-gp overexpressing multidrug resistance (MDR) cancer cell lines (MES-SA/DX5, uterine; MCF-7/ADR, breast) [27,28]. The hexane fraction of *Z. cassumunar* exhibited potent P-gp inhibitory activity with a daunomycin (DNM) IC_50_ value of 0.93 μg/mL in the MES-SA/DX5 cell line, compared with that of verapamil, which is a well-known P-gp inhibitor (IC_50_ 1.4 μM) [27], and the chloroform fraction decreased the cytotoxicity of DNM with up to a 6.56 μg/mL IC_50_ value in the MCF-7/ADR cell line (verapamil, IC_50_ 6.62 μM) [28]. In our follow-up study on the P-gp inhibitory effect of the constituent of *Z. cassumunar* [29], *trans*-3-(3,4-dimethoxyphenyl)-4-[(*E*)-3,4-dimethoxystyryl]cyclohex-1-ene (**11**) exhibited potent P-gp inhibitory activity when the IC_50_ value of DNM was decreased by more than that of verapamil. Particularly, **11** showed greater enhancement of [^3^H]-DNM accumulation and the attenuation of the [^3^H]-DNM efflux compared to those of verapamil. (*E*)-1-(3′,4′-dimethoxyphenyl)but-1,3-diene (**1**) and (*E*)-4-(2′,4′,5′-trimethoxyphenyl)but-1,3-diene (**14**), having two double bonds in the butane chain, significantly decreased the IC_50_ value of DNM, while (*E*)-4-(3′,4′-dimethoxyphenyl)but-3-en-1-ol (**2**) and (*E*)-4-(3′,4′-dimethoxyphenyl)but-3-enyl acetate (**3**) with one double bond in their structures showed a weak decrease of those. Compound **11** was discovered to be a potent modulator of P-gp activity but was isolated as a racemic mixture. However, since the pharmacological effects and pharmacokinetic properties of the racemic mixture could be different from those of the respective enantiomers, 3*S*-(3,4-dimethoxyphenyl)-4*R*-[(*E*)-3,4-dimethoxystyryl]cyclohex-1-ene (**21**) and 3*R*-(3,4-dimethoxyphenyl)-4*S*-[(*E*)-3,4-dimethoxystyryl]cyclohex-1-ene (**22**), which were optically and actively synthesized by our research group, were evaluated for their P-gp inhibitory effects in the MCF-7/ADR cell line, in which they exhibited DNM cytotoxicities with IC_50_ values of 1.44 μM and 3.19 μM [30]. Then, we identified their mechanism of action by measuring the cellular accumulation and efflux of DNM, human P-gp membrane ATPase activity, and cellular P-gp expression [31]. Compound **22** significantly changed the ratio of [^3^H]-DNM accumulation (539%) and efflux (55.4%), compared to those of **21**; thus, the in vivo application of **22** coadministrated with paclitaxel (a P-gp specific substrate) was investigated. When paclitaxel (25 mg/kg) was orally administered with 2 (5 mg/kg), its relative bioavailability was improved by 185% due to the oral exposure of paclitaxel through the inhibition of the intestinal P-gp. In our recent study, which aimed to discover an inhibitor of metastasis from phenylbutenoid and its derivatives [32], it was discovered that *trans*-3-(3,4-dimethoxyphenyl)-4-[(*E*)-3,4-dimethoxystyryl]cyclohex-1-ene (**11**) activated the nucleoside diphosphate kinase (NDPK) activity of recombinant Nm23-H1 (a tumor metastasis suppressor) in a dose-dependent manner (EC_50_, 10.7 μM). Compound **11** was shown to bind to the C-terminal of Nm23-H1 and to induce NDPK activation through allosteric conformational changes via an in silico molecular docking analysis. This compound induced morphological changes in a highly invasive breast cancer MDA-MB-231 cell line and reduced Rac1 activation via NDPK activation of Nm23-H1. This compound also suppressed the in vitro invasion and migration of MDA-MB-231 cells and in vivo metastasis in a breast cancer mouse model (a luciferase-expressing metastatic human breast cancer cell (MDA-MB-231-Luc-D3H2LN) implanted in nonobese diabetic/severe combined immune-deficient (NOD/SCID) mice). Matsuda et al. [10] elucidated the inhibitory effects of several phenylbutenoids from *Z. cassumunar* on the invasion of human fibrosarcoma HT-1080 cells. Plain I (**25**); plain III (**27**); (*E*)-1-(3′,4′-dimethoxyphenyl)but-1,3-diene (**1**); (*E*)-4-(2′,4′,5′-trimethoxyphenyl)but-1,3-diene (**14**); and β-sesquiphellandrene (**55**) have shown anti-invasive activities with the inhibition of 40.5%, 33.0%, 46.8%, 45.5%, and 29.0% at 30 μg/mL, respectively, compared with the positive control, deguelin with the inhibition of 57.9% at the same concentration. Among them, compound **14** significantly inhibited the invasion of HT-1080 cells, which was determined by very weak cytotoxicity. There was a study on the mechanisms of apoptosis induced by an abundant phenylbutenoid dimer in *Z. cassumunar*, *cis*-3-(3′,4′-dimethoxyphenyl)-4-[(*E*)-3′’’,4′’’-dimethoxystyryl]cyclohex-1-ene (**4**), toward the T-acute lymphoblastic leukemia CEMss cell line [54]. This compound exerted potent antiproliferative activity toward CEMss cells with an IC_50_ value of 7.11 μg/mL, followed by hepatocellular carcinoma (HepG2), human breast adenocarcinoma (MCF-7), human breast carcinoma (MDA-MB-231), cervical carcinoma (HeLa), and human blood mononuclear cells with IC_50_ values of 17.65, 21.28, 32.38, >50, and >50 μg/mL, respectively. The effect of **4** on the morphology of CEMss cells exhibited significant morphological changes corresponding to typical apoptosis. Compound **4** also showed a significant S phase arrest and triggered the formation of DNA fragmentation in CEMss cells. A decline in the MMP of CEMss cells by **4** was detected. After screening several proteins implicated in apoptosis induction, it was determined that **4** upregulated the Bcl-2-associated X protein (Bax), caspase-3, cytochrome c, and the second mitochondria-derived activator of caspase and downregulated B-cell lymphoma 2 (Bcl-2), heat shock protein 70, and X-linked inhibitor of apoptosis protein but did not affect caspase-8, p53, and the BH3 interacting domain death agonist. The activity of caspases-3/7 and -9 involved in the intrinsic pathway was increased in CEMss cells treated with **4**. Thus, **4** exhibited an apoptogenic property in the CEMss cell line via the intrinsic mitochondrial pathway of apoptosis induction. Previous results demonstrated that phenylbutenoids from *Z. cassumunar* possess potent anticancer activities via their preventive efficacy on MDR in chemotherapy, as well as the effects of treating cancer caused by multiple carcinogeneses, such as antiproliferative, antimetastatic, and anti-apoptotic activities in cancer.

### 3.4. Neuroprotective and Neurotrophic Activities

In an assessment of the protective effect of the ethanol extract of *Z. cassumunar* against LPS-induced neuronal cell loss and the activation of astrocytes in the hippocampus [19], pretreatment with *Z. cassumunar* extract (200 mg/kg, i.p.) in adult male Wistar rats reduced neuronal cell loss in the hippocampus and suppressed the inflammatory response by reducing the expression of the glial fibrillary acidic protein and IL-1ss in the hippocampus. The constituents of Indonesian ginger, Bangle (*Z. purpureum*), demonstrated neurotrophic effects in a series of studies [14,20]. *Trans*-3-(3′4′-dimethoxyphenyl)-4-[(E)-3′′,4′′-dimethoxystyryl]cyclohex-1-ene (**11**), and *cis*-3-(3′4′-dimethoxyphenyl)-4-[(E)-3′′,4′′-dimethoxystyryl]cyclohex-1-ene (**4**) have exerted neurotrophic effects by inducing neurite sprouting in PC12 cells and triggering neurogenesis and neurite growth and protection in primary cultured rat cortical neurons. Chronic treatment of these compounds (50 mg/kg, p.o.) enhanced hippocampal neurogenesis in olfactory bulbectomy (OBX)-induced mice and increased the number of 5-bromo-2′-deoxyuridine (BrdU)/NeuN (a neuronal marker) double-labeled cells [20]. In addition, two curcuminoids, neocassumunarins A (**48**) and B (**49**), also promoted neurite outgrowth of nerve growth factor (NGF)-mediated PC12 cells in concentrations ranging from 1 to 10 μM [14]. It was discovered that compound **48** prevents β-sheet formation in the amyloid b-protein (Aβ42). Therefore, the above results indicate that phenylbutenoid dimers have therapeutic potential for treating depression and dementia, such as Alzheimer’s disease.

### 3.5. Dermatological Activities

In our previous study on *Z. cassumunar*, one of its constituents, (*E*)-4-(3,4-dimethoxyphenyl)but-3-en-1-ol (**2**), was discovered to enhance melanogenesis through increasing upstream stimulating factor-1-mediated expressions of ERK, p38, and tyrosinase in the mouse melanoma B16 F10 cell line and to induce hyperpigmentation in brown guinea pigs in vivo [21]. In another study that aimed to identify the cosmetic potential of this plant [17], its rhizome extracts and components were evaluated for their DPPH radical scavenging, HDFa collagen secretion promotion, tyrosinase inhibition, and inhibition of LPS-induced NO production in RAW264.7 cells. The ethyl acetate extract showed greater activities in this four-assay system compared to those of the petroleum ether and chloroform extracts of the plant. Among the six constituents of the plant, the HDFa collagen secretion promoting activity of *cis*-3-(3,4-dimethoxyphenyl)-4-[(*E*)-2,4,5-trimehoxystyryl]cyclohex-1-ene (**5**) and (1*E*,4*E*,6*E*)-1,7-bis(4-hydroxyphenyl)-1,4,6-heptatrien-3-one (**46**), the tyrosinase inhibiting activity of **46**, and the NO production inhibiting activities of 1-feruloyloxycinnamic acid (**53**) and bisdemethoxycurcumin (**47**) were found. Therefore, the *Z. cassumunar* extract or its constituents have potential applications in the develop natural cosmetic and pharmaceutical products for preventing/treating hypopigmentation, skin aging, or dermatitis.

### 3.6. Antifungal and Antibacterial Activities

The oil of the rhizomes of *Z. cassumunar* was reported to have high antifungal activity (zone of inhibition: 11.7‒15.7 mm) against five strains of yeasts, *Saccharomyces cerevisiae*, *Cryptococcus neoformans*, *Candida albicans*, *Candida tropicalis,* and *Torulopsis glabrata* [23]. *Z. cassumunar* oil containing 32 vol % of terpinen-4-ol as its major constituent exhibited antimicrobial activity against a wide range of bacteria (Gram-positive: *Staphylococcus aureus* ATCC 29737, *Streptococcus pyogenes*, *Bacillus subtilis* ATCC6633, *Streptococcus epidermidis* ATCC 12228, and *Propionibacterium acnes*; Gram-negative: *Escherichia coli* ATCC 10536, *Salmonella typhi*, *Pseudomonas aeruginosa* ATCC 25619, *Klebsiella pneumoniae* ATCC 10031, and *Proteus vulgaris*) with minimum bactericidal concentrations ranging from 0.62 to 2.5 vol % [24]. Antifungal activity against dermatophytes (*Epidermophyton floccosum*, *Microsporum gypseum*, *Trichophyton mentagrophytes*, and *Trichophyton rubrum*) and yeasts (*Candida albicans* and *Cryptococcus neoformans*) by the oil has presented the minimum fungicidal concentration ranging from 0.31 to 0.62 vol %. The hydrodistilled essential oil of cassumunar ginger (*Z. montanum*) containing sabinene, terpinen-4-ol (9.0–31.3%), γ-terpinene, β-phellandrene, and (*E*)-1-(3′,4′-dimethoxyphenyl)buta-1,3-diene as major components was evaluated against eight pathogenic bacteria (Gram-positive: *Staphylococcus aureus* (MTCC 96), *Staphylococcus epidermidis* (MTCC 435), and *Streptococcus mutans* (MTCC 890); Gram-negative: *Klebsiella pneumoniae* (MTCC 109), *Pseudomonas aeruginosa* (MTCC 741), *Escherichia coli* (MTCC 723), *Escherichia coli* (DH5*𝛼*), and *Salmonella typhimurium* (MTCC 98)) and exhibited good antibacterial activity with minimum inhibitory concentration values ranging from 125 to 500 μg/mL), which is an indication the lowest for *S. typhimurium* [22]. The oil also showed antifungal activity (250 μg/mL) against two fungal strains (*Candida albicans* (ATCC 14053) and *C. albicans* (MTCC 1637)). The allelopathic activity of the oil on germination and seedling growth of lettuce seed was also demonstrated.

## 4. Conclusions

This review presented a comprehensive report of the phytochemicals and bioactivities of *Z. cassumunar*. As traditional uses, the rhizomes of this plant have been widely used in different countries in Southeast Asia for the treatment of inflammation, pain, rheumatic arthritis, asthma, and skin trouble. Since 1970s, many studies on the phytochemicals and bioactivity of *Z. cassumunar* have been conducted in order to establish the scientific fundamental facts and evidence of such traditional medicinal uses. Phenylbutenoids and curcuminoids have been mainly isolated from *Z. cassumunar* as a single compound, and its essential oil containing monoterpenoids, sesquiterpenoids, and phenylbutenoids has been assessed primarily for its pharmacological and cosmeceutical use. The extracts, solvent fractions, and constituents of this plant have been discovered to have multiple bioactivities, such as antioxidant, anti-inflammatory (including joint and respiratory inflammation), anticancer, neuroprotective/neurotrophic, and dermatological properties. Therefore, we hope that this review will help to establish experimental design by providing the scientific information for future research and to discover more new medicinal values of *Z. cassumunar*.

## Figures and Tables

**Table 1:** Bioactivities of extracts, fractions, and constituents of *Z. cassumunar*.

Extracts, Fractions, or Compounds	Bioactivities	Cell Lines or Models	Ref.
Essential oil	Antioxidant	ABTS scavenging activity; H_2_O_2_ scavenging activity in U937 cells	[38]
		SOD enzyme activity in rat induced by HFD	[44]
	Anti-inflammatory	Carrageenin-induced hind-paw edema test in rats	[40]
		LPS-induced NO production in RAW264.7 cells	[45]
	Antifungal	*Saccharomyces cerevisiae*, *Cryptococcus neoformans*, *Candida albicans*, *Candida tropicalis*, and *Torulopsis glabrata*	[23]
		*Epidermophyton floccosum*, *Microsporum gypseum*, *Trichophyton mentagrophytes*, *Trichophyton rubrum*, *Candida albicans*, and *Cryptococcus neoformans*	[24]
		*Candida albicans* (ATCC 14053) and *C. albicans* (MTCC 1637)	[22]
	Antimicrobial	Gram-positive: *Staphylococcus aureus* ATCC 29737, *Streptococcus pyogenes*, *Bacillus subtilis* ATCC6633, *Streptococcus epidermidis* ATCC 12228, and *Propionibacterium acnes*; Gram-negative: *Escherichia coli* ATCC 10536, *Salmonella typhi*, *Pseudomonas aeruginosa* ATCC 25619, *Klebsiella pneumoniae* ATCC 10031, and *Proteus vulgaris*	[24]
		Gram-positive: *Staphylococcus aureus* (MTCC 96), *Staphylococcus epidermidis* (MTCC 435), and *Streptococcus mutans* (MTCC 890); Gram-negative: *Klebsiella pneumoniae* (MTCC 109), *Pseudomonas aerugenosa* (MTCC 741), *Escherichia coli* (MTCC 723), *Escherichia coli* (DH5𝛼), and *Salmonella typhimurium* (MTCC 98)	[22]
Methanol extract	Anti-inflammatory	Carrageenin-induced hind-paw edema test in rats; acetic acid-induced vascular permeability and writhing test in mice	[46]
Ethanol extract	Antioxidant	SOD enzyme activity in rat induced by HFD	[44]
	Anti-asthma	PMA-induced mucin production in NCI-H292 cells	[47]
	Neuroprotective	LPS-induced neuronal cell loss and astroglial activation within the hippocampus using adult male Wistar rats	[19]
Ether fraction	Anti-inflammatory	Carrageenin-induced hind-paw edema test in rats; acetic acid-induced vascular permeability and writhing test in mice	[46]
Hexane fraction	Anti-inflammatory	Carrageenin-induced hind-paw edema test in rats; acetic acid-induced vascular permeability and writhing test in mice	[46]
		TPA-induced ear edema in rats	[48]
		LPS-induced NO production in RAW264.7 cells	[45]
	Antioxidant	H_2_O_2_ scavenging activity; α-glucosidase inhibition	[43]
	Anticancer	Cytotoxicity of DNM in MES-SA/DX5 and MCF-7/ADR cell lines	[27,28]
Chloroform fraction	Antioxidant	DPPH scavenging activity	[43]
	Anticancer	Cytotoxicity against A549 and SNU-638 cell lines	[25]
		Cytotoxicity of DNM in MES-SA/DX5 and MCF-7/ADR cell lines	[27,28]
Ethyl acetate fraction	Antiaging, skin whitening, and anti-inflammation	DPPH scavenging, HDFa collagen secretion, tyrosinase inhibition, and LPS-induced NO production in RAW264.7 cells	[17]
Phenylbutenoid-rich fraction	Anti-inflammatory	LPS-induced NO production in RAW264.7 cells	[45]
**1**	Anti-inflammatory	EPP, AA, TPA, or carrageenan-induced ear edema in rats; collagen, ADP, AA, or PAF-induced platelet aggregation	[42]
		LPS-induced PGE2 production in RAW264.7 cells	[9]
		LPS-induced NO production in RAW264.7 cells	[13,45]
		LPS-induced PGE2 level and COX-2 expression in human dental pulp cells	[49]
	Anticancer	Cytotoxicity against HT-1080 cell line	[8]
		Cytotoxicity of DNM in MCF-7/ADR cell lines	[29]
		Invasion of HT-1080 cells	[10]
**2**	Anti-inflammatory	TPA-induced ear edema in rats	[48]
		LPS-induced NO production in RAW264.7 cells	[45]
	Chondroprotective effect	Cytokine-induced cartilage degradation in explant culture	[50]
		Cytokine-induced up-regulation of catabolic genes involved in joint erosion in SW982 cells	[51]
	Anti-asthma	Pro-MMP-9 by house dust mite allergens; MMP-9 expression in PMA-stimulated NCI-H292cells	[52]
		Molecular docking and molecular dynamics simulations with 5-LO enzyme	[53]
	Melanogenic effect	Melanin synthesis in B16F10 cells and human primary melanocytes; USF-1-mediated tyrosinase expression; hyperpigmentation in brown guinea pigs.	[21]
**3**	Anti-inflammatory	TPA-induced ear edema in rats	[48]
		LPS-induced NO production in RAW264.7 cells	[45]
**4**	Anti-inflammatory	TPA-induced ear edema in rats	[48]
		LPS-induced PGE2 level and COX-2 expression in human dental pulp cells	[49]
	Anticancer	Antiproliferative activity toward CEMss, HepG2, MCF-7, MDA-MB-231, and human blood mononuclear cell lines; apoptosis in CEMss cells via induction of p53-independent mitochondrial signaling pathway	[54]
	Neurotrophic	Induction of neurite sprouting in PC12 cells; neurogenesis and neurite growth and protection in primary cultured rat cortical neurons; hippocampal neurogenesis in OBX-induced mice	[20]
**5**	Anti-inflammatory	TPA-induced ear edema in rats	[48]
	Chondroprotective effect	Cytokine-induced cartilage degradation in explant culture	[50]
	Collagen promoting	HDFa collagen secretion	[17]
**6**	Anti-inflammatory	TPA-induced ear edema in rats	[48]
		LPS-induced PGE2 level and COX-2 expression in human dental pulp cells	[49]
**8**	Anti-inflammatory	Carrageenin-induced hind-paw edema test in rats; acetic acid-induced vascular permeability and writhing test in mice	[46]
		Carrageenin-induced hind-paw edema test in rats	[40]
**9**	Anti-inflammatory	LPS-induced NO production in mouse peritoneal macrophages	[13]
**11**	Anti-inflammatory	PGE2 production in the LPS-stimulated mouse macrophage RAW264.7 cells	[9]
	Anticancer	Cytotoxicity against A549, Col2, SNU-638, and HT-1080 cell lines	[8]
		Growth inhibition and induction of G1 phase cell cycle arrest in A549 cells	[26]
		Cytotoxicity of DNM in MCF-7/ADR cell line	[29]
		Activation of NDPK activity of recombinant human Nm23-H1 and cellular NDPKs in MDA-MB-231 cells; in vitro invasion and migration of MDA-MB-231 cells; in vivo metastasis in MDA-MB-231-Luc-D3H2LN mice	[32]
	Neurotrophic	Induction of neurite sprouting in PC12 cells; neurogenesis and neurite growth and protection in primary cultured rat cortical neurons; hippocampal neurogenesis in OBX-induced mice	[20]
**14**	Anti-inflammatory	PGE2 production in the LPS-stimulated mouse macrophage RAW264.7 cells	[9]
		LPS-induced NO production in mouse peritoneal macrophages	[13]
	Anticancer	Cytotoxicity of DNM in MCF-7/ADR cell lines	[29]
		Invasion of HT-1080 cells	[10]
**17**	Anti-inflammatory	Carrageenin-induced paw edema in rats; Carrageenin-induced rat pleurisy; adjuvant-induced arthritis	[7]
	Analgesic	Acetic acid-induced writhing response in mice; tail-flick test in rats	
	Antipyretic	Yeast-induced hyperthermia in rats	
**18**	Anti-inflammatory	PGE2 production in the LPS-stimulated mouse macrophage RAW264.7 cells	[9]
	Anticancer	Cytotoxicity against A549, Col2, SNU-638, and HT-1080 cell lines	[8]
**21**	Anticancer	Cytotoxicity of DNM in MCF-7/ADR cell lines	[30]
**22**	Anticancer	Cellular accumulation and efflux of DNM in MCF-7/ADR cell lines; in vivo application of paclitaxel and co-administration using male Sprague Dawley rats	[30,31]
**25, 27, 55**	Anti-inflammatory	LPS-induced NO production in mouse peritoneal macrophages	[13]
	Anticancer	Invasion of HT-1080 cells	[10]
**30, 42**	Anti-inflammatory	LPS-induced NO production in mouse peritoneal macrophages	[13]
**43, 44**	Antioxidant	Thymocytes under H2O2-iduced oxidative stress	[42]
**46**	Collagen promoting	HDFa collagen secretion	[17]
	Skin whitening	Tyrosinase inhibition	[17]
**47**	Anti-inflammatory	LPS-induced NO production in Rwa264.7 cells	[17]
**48**	Neurotrophic	Neurite outgrowth of NGF-mediated PC12 cells	[14]
**49**	Anti-inflammatory	LPS-induced NO production in mouse peritoneal macrophages	[13]
	Neurotrophic	Neurite outgrowth of NGF-mediated PC12 cells	[14]
**59, 60**	Anti-inflammatory	Carrageenin-induced hind-paw edema test in rats	[40]

## Data Availability

Not applicable.
